# A Report from the Central Committee of the Society for Propagating Vaccination, on the Number of Children Vaccinated in France during the Years 1818 and 1819

**Published:** 1821-11

**Authors:** 


### CRITICAL ANALYSES

OF

### RECENT PUBLICATIONS, IN THE DIFFERENT BRANCHES OF MEDICINE AND SURGERY,

### In the Literature of Foreign Nations.

---

Πατρίδες ἄρα
Ανδράσιν, ὦ πατραῖς ἄνδρες, ἀγαλλόμεθα.

---

*Rapport du Comité Central de Vaccine, sur les Vaccinations Pratiquées en France pendant les années* 1818 *et* 1819;—*or, a Report from the Central Committee of the Society for propagating Vaccination, on the Number of Children vaccinated in France during the years* 1818 *and* 1819. Paris, 1821. pp. 132. (For private circulation.)

"THEY certainly manage these matters better in France."—The means adopted by our transmarine neighbours for the propagation of vaccination in their own country, are admirably calculated to attain that desirable object. Foremost amongst those measures we must place those annual festivals and public meetings of the Vaccine Society, at which every class of persons is admitted; and where, after a plain and gratifying statement of the success of vaccination during the preceding year, medals and pecuniary rewards are distributed, with suitable eulogiums, to those individuals, whether of the medical profession or otherwise, who have most distinguished themselves for the zeal with which they promoted vaccination. One of these solemn festivities, so honourable to the author of the great discovery which it is intended to commemorate, lately took place in Paris, agreeably to custom; and it is now our pleasing duty to record the occurrence, at the same time that we shall endeavour to give our readers an account of its result.

On Saturday, the 5th of May, 1821, Baron CAPELLE, counsellor of state, charged with the administration of public hospitals and other medical charities, held, and presided at, a general meeting of the Central Vaccine Society, in the great library of the Faculty of Medicine. Several professors of that faculty, many physicians and surgeons, clergymen of the catholic and reformed religions, and a vast concourse of pupils and people, attended the meeting. Baron Capelle opened the business of the day, by calling the attention of the meeting to the increasing benefits accruing to society from the discovery of vaccination; a discovery, than which no other called more loudly for the gratitude of mankind. The Baron's description of the obligation we owe to JENNER is both neat and appropriate:

"De tous les bienfaits consacrés par la reconnaissance publique, il en est peu qui méritent autant cet hommage que celui dont vous êtes les savans propagateurs: en lui tout est vrai, tout est avantageux; la victoire n'est acheté par aucun sacrifice, la découverte par aucun erreur; rien n'y ressemble à ces conceptions hasardées dans lesquelles la sagesse de l'expérience est souvent sacrifiée à la vanité des innovations. La vaccine a déjà répondu par vingt-cinq ans de succès aux préjugés qui la combattaient; et l'espèce humaine, secourue par elle contre un des plus terribles fléaux qui la menacent, trouve dans sa propre conversation un témoignage qui rend désormais toutes les critiques impuissantes et tous les élogés superflus."

The Baron concluded by observing, that much yet remained to be done, and that it was important "de ne point s'arreter tant qu'il reste des progrés à obtenir."

Professor CHAUSSIER, president of the central committee of the Vaccine Society, whose efforts in encouraging vaccination have been as successful as they have been constant, next addressed the meeting; and observed, that, from the multiplied experiments and numerous observations made during more than twenty years, it had been ascertained, in the most positive manner, that vaccination was most efficacious in preventing the small-pox, and might possibly become the means of completely eradicating that disease; that, in France, no effort was wanting to promote so desirable an end; and that the central committee, with a view to that object, kept up a constant correspondence in every, even the most remote, part of the kingdom, and distributed vaccine matter with a praise-worthy profusion. It was but lately that a large quantity of that matter was forwarded to China, as a present from the French king to the sovereign of that vast empire, in which the small-pox had, last year, committed great ravages. The government has determined that pecuniary rewards shall be annually distributed to the most zealous vaccinators; and, to second the philanthropic views of the administration, the prefects of the departments have established special committees in every town, charged to propagate vaccination, through the assistance of expert physicians, surgeons, and pastors. Mr. Chaussier acknowledged at the same time, that, notwithstanding the example of sovereigns, who submitted their own infant children to the operation, and the expressed conviction of men conspicuous for their talents and philanthropy, vaccination had not yet reached that degree of universality which the welfare of mankind still imperiously demands.

The Professor then went on to investigate the different causes which have retarded the progress of vaccination; and successfully combatted all the objections raised against it. In doing this, the Professor avails himself of every fact and argument urged by the National Vaccine Establishment, the College of Surgeons, and Sir Gilbert Blane, in England, for the same laudable purpose, in their respective documents; copies of which had been transmitted to the Professor.

We are, however, of opinion, like many others, that coercive means, more or less severe, according to the spirit of the laws, ought to be employed by every government, in order to ensure the universal adoption of a measure, the prejudices against which it would be a waste of time to endeavour to combat by means of plain reasoning only and fair council.

"Le raisonnement (observes Professor Chaussier,) ne fait point d'impression sur ceux qui ne sont pas accoutemés à réfléchir, à observer, à en suivre les conséquences: les instructions, les moyens de persuasion, échouent contre l'entêtement et l'obstination."

This conviction has induced several of the prefects of the departments to issue orders that no child should be admitted to partake of the benefits of any of the public charities or establishments, that shall not appear to have been vaccinated. In imitation of this praiseworthy conduct on the part of the French magistrates, the founders of the Royal Metropolitan Infirmary for Children, in London, have resolved that the medical officers of that institution shall be empowered to withhold the benefits of that charity from those children whose mothers shall object to vaccination, without any very solid reason. Had some such resolution as this been adopted by the officers of every other medical or philanthropic institution in England, the prejudices of the major part of the lower classes of society against the salutary measure under our consideration, would, ere now, have been greatly diminished, if not wholly removed. The great legislator of the Jews, knowing how conducive to the health of his people it was to enforce cleanliness, among other measures of hygiene, made it a precept that every child should, at its birth, be subjected to a certain operation, the purport of which was of very trifling importance compared to that for which vaccination might be made obligatory on every parent by a law of the land.

Professor Chaussier having concluded his address, the report of the central committee of vaccination, on the number of children vaccinated during the years 1818 and 1819, was read by Mr. Husson, the perpetual secretary of the society. This document presents an *ensemble*

of the plans uniformly adopted in most parts of France for the propagation of vaccine, which deserves our best consideration. These plans appear so admirably calculated to attain the desired end, that we are surprised at their not having been more generally followed even in France. In those departments where the prefect has deemed it his duty to extend his authority to the practice of vaccination, the best effects have ensued, and the numerical results from the occurrence of natural small-pox, compared to those derived from the inoculation of cow-pox, are by far more favourable to the latter. Admonitory as well as compulsory instructions have been published; persons employed in public capacities have been readily persuaded to have their offspring vaccinated; their servants, being soon convinced of the utility of that practice, hastened, with little hesitation, to imitate their masters; while the shopkeeper, the petty tradesman, the independent manufacturer, and the husbandman, tacitly availed themselves of a salutary measure, which they saw adopted, with so much apparent earnestness and benefit, by their superiors.

In every small town, borough, or village, vaccine matter has been deposited, from the best sources, in the hands of the clergyman, the medical practitioner, or, in the absence of them, the principal housekeeper. Ambulating vaccinators, on whose skill and integrity the prefect could rely, have been appointed to journey from one place to another, and either to vaccinate themselves, or teach the individuals above mentioned to practise with safety and dexterity that operation. Forms of registers have been drawn up, and copies of them furnished to all those who are to perform vaccination; and, lastly, sub-committees have been appointed by the prefect in the principal towns of his district, charged to collect all the information from minor places, with a view to draw up an annual report, which is forwarded to the central committee at Paris. To encourage the tardy, and to reward the zealous,—premiums, some pecuniary, others honorary, have been established, and apparently with the best effect.

There is a class of persons in France, to whom the operation of vaccinating children can with propriety be confided,—namely, that of midwives; for in that country, where midwifery is held in proper consideration, and its importance fully felt, no woman is allowed to practise as a midwife who has not had a regular education at the great establishment of the Maternité, at Paris; where, amongst other useful qualifications, the female pupils are instructed in performing the operation of vaccination. This latter circumstance affords the French a greater facility than we possess in this country for propagating the cow-pox, as the only means of extirpating the dreadful contagion against which it is known to act specifically. There is now scarcely a village of any consequence in France in which there is not a resident midwife, qualified in the manner we have just described, and who has, moreover, been brought up to love and cherish the salutary practice to which our efforts, in the present article, have been directed.

We have no room to enter further into the details of administration relative to the propagation of vaccination in France, contained in the first part of the Report; and proceed, therefore, to the second part, or division, of this important document. This, properly speaking, is more interesting even than the former to medical men, as it professes to give an account of the principal medical facts connected with vaccination, with which the central committee have become acquainted through their numerous correspondents.

The first of these facts relate to the irregularity observed in the progress of the pock after inoculation. Cases have been known to occur in which the pustule has furnished, after the fourth day, well-formed lymph, from which other children have been vaccinated, when the pustule was found to have lost that degree of precocity. Instances where the first appearance of the insertion having succeeded, manifested itself on the seventh, eighth, ninth, and as late as the thirteenth day, have occurred on various well-authenticated occasions.

It is now placed beyond doubt that the vaccine matter will occasionally give rise to the spontaneous eruption of many more pustules in different parts of the body, the matter of which is perfectly good for inoculation. Of this fact we have ourselves been witnesses in more than one instance; and it goes far, in our opinion, to show that there is a general action produced by the vaccine virus on the constitution, besides the local effect which arises from it.

Thirteen medical men have seen examples of vaccination proving the means of curing other eruptive diseases, the *crusta lactea* in particular; and it stands recorded in the present Report, upon unimpeachable authority, that the inoculated cow-pox has effectually cured scrofulous swellings, ophthalmia, chincough; and has appeared to exert a beneficial influence in cases of epilepsia, hemiplegia, and sciatica.

The Report is full of the experiments that have been repeatedly made with a view to ascertain the efficacy of vaccination in subjects who were subsequently inoculated with the small-pox, or exposed to its contagion. The result has been uniformly favourable to the doctrine of the immortal Jenner. Four instances are detailed of mothers, attacked with the small-pox, safely suckling their infants that had been vaccinated.

All the above facts, many of which have been observed before, were confirmed during the prevalence of varioloid epidemics, which have of late years partially ravaged some of the provincial districts of France. Mr. Lejeune, for instance, relates that, in the village of Claye, (department of l'Aisne,) of thirteen children, one only, that had been vaccinated, resisted the small-pox by which the other twelve were attacked, and of which four of the twelve died.

The influence which the cow-pox seems to exert over the small-pox has been strongly illustrated in those individuals who, having been vaccinated after exposure to the variolous contagion, have experienced a disease extremely mild in all its characters.

The Report lays a great stress on the comparative rarity of the small-pox in places where vaccination has been much and generally practised.

It then goes on to notice the pretended cases of failure, and observes that "Lorsqu'on analyse tous ces faits, on arrive toujours à un des résultats suivans: ou la vaccination quoique pratiquée n'a été suivi d'aucun développement, ou l'opération a produit une vaccine fausse et non préservative, ou, comme nous l'avons vu précèdemment, la variole a éclaté pendant le cours de la vaccine, ou enfin on a pris pour une petite vérole contagieuse, une éruption qui a avec elle quelques points de ressemblance, et que pour cette raison on peut appeler *varioloïde*."

Some few of the cases themselves are next given, and carefully analyzed; and we hesitate not in saying, that the conclusions of the reporter seem correct.

We cannot conclude our account of this very instructive pamphlet without quoting, in the original language, the parallel drawn up, with great care and truth, between the real small-pox and the chicken-pox; respecting whose identity or dissimilarity so much has been said and written in this country.

"Nous ne parlerons pas des symptômes par lesquels s'annoncent les deux maladies: ces symptômes, généralement communs aux maladies éruptives aiguës, ne diffèrent que par leur plus ou moins d'intensité, et cette différence n'est pas encore assez constamment uniforme pour qu'elle puisse seule être caractéristique; mais nous nous arrètons à la fièvre qui se manifeste dans les deux affections, et nous ferons observer que, dans la petite vérole vraie, elle dure trois ou quatre jours; que, si elle diminue ou cesse à l'apparition des boutons, c'est pour reparaître avec la suppuration, entre le neuvième et le trezième jour: lorsque, dans la petite vérole bâtarde ou volante, elle ne dure qu'un jour ou deux, et est quelquefois à peine sensible.

"Dans la vraie, l'éruption ne commence qu'après trois jours de fièvre, et se montre d'abord au visage, au cou, à la poitrine, et successivement aux autres parties du corps; les boutons augmentent insensiblement, et n'ont acquis leur grosseur qu'au bout de quatre ou cinq jours.

"Dans la fausse, l'éruption paraît tout-à-coup à la fin du premier jour, quelquefois du second, et rarement le troisième; elle se fait presque simultanément sur les différentes parties du corps, et sans cet ordre régulier que suivent, à leur sortie, les boutons de la petite vérole.

"Dans la vraie, les pustules, une fois formées, présentent un léger enfoncement au milieu de leur sommet; d'abord rouges, elles blanchissent ensuite, et sont environnées d'une aréole de couleur rosée.

"Dans la fausse, elles sont sphériques à leur sommet.

"Dans la vraie, ces pustules sont remplies d'une sérosité qui se transforme en une matière purulente, exhalant une odeur nauséabonde particulière.

"Dans la fausse, elles ne contiennent, même au point de leur plus grande maturité, qu'une lymphe blanchâtre et transparente.

"Dans la petite vérole, le dessèchement ne commence que le onzième jour de la maladie.

"Dans la vérolette, les pustules laissent transsuder l'humeur qu'elles contiennent, quelquefois deux ou trois jours après leur sortie; elles se flétrissent, se dessèchent et tombent en écailles à la fin du sixième, huitième, ou dixième jour.

"Dans la petite vérole, l'éruption, la suppuration et la dessiccation forment trois périodes successives, bien distinctes l'une de l'autre par la durée, la première de trois jours, les deux autres de cinq jours chacune, ou environ.

"Dans la vérolette, ces trois périodes semblent se confondre, soit parce que leur durée n'excède pas six ou huit jours, soit parce qu'elles se manifestent à-la-fois, et qu'il résulte de cette intermixtion que beaucoup de pustules sont déjà sèches sur différentes parties du corps, pendant qu'on en voit d'autres en pleine maturité ou même naissantes.

"Dans la petite vérole, il survient avant l'exsiccation un gonflement général de la peau, sensible sur-tout à la face, aux mains, et quelquefois tellement marqué aux paupières, que les malades peuvent être momentanément privés de voir la lumière.

"Dans la vérolette, le desséchement n'est précédé d'aucun gonflement, ou du moins il est presque insensible.

"Un des caractères les plus notables de la petite verole, est cette rougeur prononcée que conservent pendant long-temps, sur la figure, les individus qui viennent d'en être atteints, tandis que les marques laissées sur la peau par la vérolette, sont à peine rouges, ou que les rougeurs, si elles sont apparentes, ne tardent pas à s'effacer.

"Les cicatrices que laisse la petite vérole, sont plus ou moins profondes; leur surface est inégale.

"Dans la vérolette, elles n'offrent point d'inégalités, et sont plus ou moins lisses ou unies; différences qui doivent résulter de celles que présente l'état inflammatoire dans l'une et l'autre éruption.

"Enfin, la petite vérole, même discrète et de moyenne intensité, est une maladie qui dure quinze ou vingt jours, et dont l'issue est quelquefois funeste.

"La vérolette, au contraire, se termine quelquefois en cinq ou six jours, ou huit ou dix au plus, et personne n'en a jamais été victime.

"Telles sont les différences essentielles qui établissent entre les deux maladies une ligne évidente de démarcation, à l'aide de laquelle on doit nécessairement se garantir de toute erreur; différences qui, abstraction faite de la nécessité qu'il y a d'en savoir bien faire la distinction, pour être en état de répondre aux attaques dirigées contre la vaccine, ont toujours été importantes à reconnaître, puisqu'on a vu autrefois des individus, trompés par l'idée qu'ils avaient d'avoir été soumis à l'invasion de la petite vérole, se dispenser de chercher dans l'inoculation de cette maladie un préservatif contre ses coups meurtriers, et payer de leur vie cette fatale persuasion."

But, after all, where is the triumph of the antivaccinist, supposing even that all the pretended cases of reported small-pox after regular and perfect vaccination were true? Have not the advocates of the Jennerian discovery one million for every such case, in which vaccine has proved the best and the surest protection against that terrible scourge the small-pox? Nay, is it not agreed on all hands, that, even where the natural small-pox has actually invaded a person who had been regularly vaccinated, (and such instances, well made out, are few in number,) the disease has run its course in a milder manner than it is known to do under different circumstances, and has seldom, if ever, proved fatal to the patient? Are there not at this moment whole districts,—nay, kingdoms,—altogether free from the variolous pestilence, since the propagation of the Jennerian discovery? and what more can man expect from the work of man?

The Report is signed by Chaussier, president; Corvisart, Delasteyrie, Doussin-Dubreuil, Hallé, Huzard, Jadelot, J. J. Leroux, Parfait, Pinel, Salmade, Duchanoy, Alibert, Bourdois, Guerbois; and Husson, secretary; and is followed by an ordinance of the minister of the interior, decreeing the distribution of one premium of 3000, two of 2000, and three of 1000 francs each, to the individuals who most distinguished themselves in propagating vaccination; and the awarding of gold and silver medals, for the same reason, to upwards of two hundred persons, all of the medical profession, with one single exception.

A voluminous Table follows the Report, containing the names of the departments, their population, the number of births, of those vaccinated, those attacked with the small-pox, those rendered infirm or disfigured by that disorder, those who died of it; the proportion of vaccinations to the number of births, the names of the operators; the amount of the expenditure for promoting vaccination; and general observations. The total result taken from this Table, occupying twenty-six pages, may be collected from the following recapitulation:

| | Population | Births. | Vaccinations. | Subjects who had the Small-pox. | Deformed or Infirm from it. | Died of Small-pox. | Proportion of Vaccinations to Births. |
|---|---|---|---|---|---|---|---|
| 81 Departs. 1818. | .......... | 722,589 | 442,945 | 43,939 | 2,392 | 6,171 | 1 in 1⅓ |
| 81 Departs. 1819. | .......... | 835,550 | 425,510 | 52,036 | 4,264 | 6,586 | 1 in 2 |
| Total.... | 27,135,119 | 1,558,139 | 868,455 | 95,975 | 6,656 | 12,857 | 1 in 1½ |

Amount of expences in the two years ...... { 1818.... 95,292,57 / 1819....101,914,33 } 198,206,90 fr.

